# Complete disappearance of ductal carcinoma *in situ* after neoadjuvant therapy in HER2-positive breast cancer: a case report

**DOI:** 10.1097/MS9.0000000000005250

**Published:** 2026-06-15

**Authors:** Qi Li, Chenghao Liu, Chaohua Hu

**Affiliations:** a Department of Thyroid and Breast Surgery, Xiaogan Hospital, School of Medicine, Wuhan University of Science and Technology, Xiaogan, Hubei, China; b School of Medicine, Wuhan University of Science and Technology, Wuhan, Hubei, China

**Keywords:** breast cancer, ductal carcinoma *in situ* with microinvasion, lymph node metastasis, microcalcifications, neoadjuvant therapy

## Abstract

**Introduction and importance:**

Ductal carcinoma *in situ* (DCIS) is traditionally considered poorly responsive to neoadjuvant therapy (NAT) because the intact myoepithelial layer and basement membrane may limit drug penetration. Recent studies, however, suggest that some DCIS components accompanying invasive breast cancer may regress after NAT, particularly in biologically aggressive subtypes.

**Case presentation:**

A 51-year-old woman presented with a right breast mass, clustered microcalcifications, biopsy-proven axillary nodal metastasis, and a suspicious subclavicular node. Core needle biopsy of the breast showed high-grade DCIS with focal suspicious microinvasion and an HR-negative/HER2-positive phenotype. She received anthracycline- and taxane-based NAT with anti-HER2 therapy, followed by a right modified radical mastectomy and nodal dissection. Final pathology demonstrated no residual invasive carcinoma, no residual DCIS in the breast, and no residual nodal disease, consistent with a pathological complete response.

**Clinical discussion:**

This finding should not be interpreted as definitive evidence that DCIS itself is chemosensitive. In the present case, the disappearance of the DCIS component may have resulted from a bystander effect after the disruption of the ductal barrier by microinvasive or unsampled invasive disease. In addition, persistent post-NAT microcalcifications on mammography did not indicate viable residual tumor, highlighting the risk of overtreatment when the surgical extent is determined primarily by residual calcifications.

**Conclusion:**

This case underscores three clinically relevant points: nodal metastasis in biopsy-diagnosed DCIS/ductal carcinoma *in situ* with microinvasion should prompt careful reassessment for occult invasion; disappearance of DCIS after NAT is hypothesis-generating rather than proof of direct chemosensitivity; and residual microcalcifications alone should not determine the surgical extent.

## Introduction

Breast cancer is a heterogeneous disease, and its pathological classification is primarily based on the expression of estrogen receptor (ER), progesterone receptor (PR), and human epidermal growth factor receptor 2 (HER2)^[^1,2^]^, which have important implications for prognosis and treatment. Ductal carcinoma *in situ* (DCIS) is a neoplastic lesion confined to the terminal duct-lobular unit without breaching the basement membrane. Because DCIS is surrounded by an intact myoepithelial layer and basement membrane, which together form a natural drug-tissue barrier, it has traditionally been regarded as poorly responsive to cytotoxic agents. Accordingly, neoadjuvant therapy (NAT) is not indicated for pure DCIS. Nevertheless, recent studies have suggested that, in selected molecular subtypes such as HER2-positive and triple-negative disease, DCIS accompanying invasive carcinoma may disappear after NAT, although the underlying mechanism remains uncertain.

HIGHLIGHTS

- Nodal metastasis in biopsy-diagnosed ductal carcinoma *in situ* (DCIS)/ductal carcinoma *in situ* with microinvasion should prompt reassessment for occult invasive disease.
- Disappearance of DCIS after neoadjuvant therapy (NAT) may reflect a bystander effect rather than direct chemosensitivity.
- Persistent post-NAT microcalcifications do not necessarily indicate viable residual tumor.

Here, we report a patient with HR-negative/HER2-positive breast cancer whose core needle biopsy showed high-grade DCIS with focal suspicious microinvasion, accompanied by lymph node metastasis. After dual HER2-targeted NAT, the patient achieved pathological complete response (pCR), and the DCIS component was no longer detectable in the surgical specimen. Through this case and a focused review of the literature, we discuss three clinically relevant questions: (1) How should DCIS/ductal carcinoma *in situ* with microinvasion (DCISM) with lymph node metastasis be interpreted diagnostically? (2) Does the disappearance of DCIS after NAT reflect true drug sensitivity or a bystander effect? and (3) What is the clinical significance of residual microcalcifications after NAT? This diagnostic pitfall may offer important lessons for individualized treatment planning.

## Case presentation

A 51-year-old woman was admitted on 7 October 2024 because of a right breast mass that had been present for more than 1 month. She had no relevant medical history. Physical examination revealed a firm right breast mass measuring approximately 6 × 5 cm, with indistinct borders and limited mobility. Enlarged lymph nodes were palpable in the right axilla.

**Figure 1.**
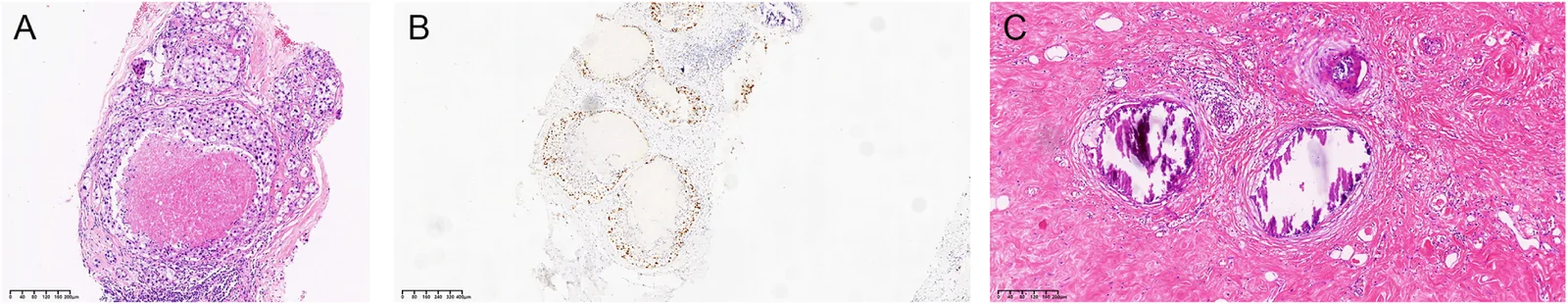
Preoperative and postoperative pathological comparison (breast). (A) Preoperative breast core needle biopsy showing high-grade ductal carcinoma *in situ* (DCIS) with focal microinvasion (HE, ×100). (B) Ki-67 immunohistochemical staining showing a proliferation index of 40% (IHC, ×50). (C) Postoperative breast specimen demonstrating pathological complete response (pCR), with no residual tumor cells, including the DCIS component (HE, ×100).

**Figure 2.**
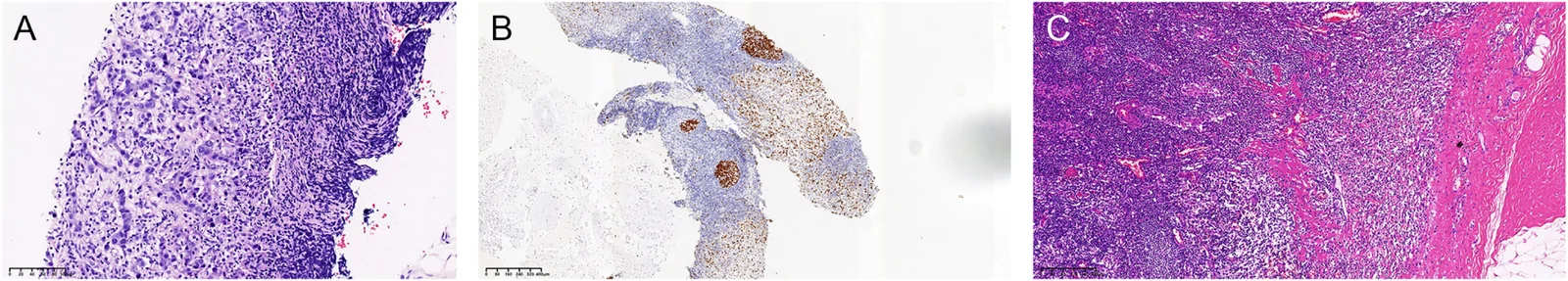
Preoperative and postoperative pathological comparison (axillary lymph nodes). (A) Pre-treatment core needle biopsy of an axillary lymph node showing metastatic carcinoma consistent with breast origin (HE, ×200). (B) Ki-67 immunohistochemical staining showing a proliferation index of 50% (IHC, ×50). (C) Post-treatment axillary lymph node dissection specimen showing no evidence of metastatic carcinoma (HE, ×100).

Breast and axillary ultrasonography performed at another hospital on 2 October 2024 showed a solid nodule in the right breast (BI-RADS category 4C) and abnormal right axillary lymph nodes. After presentation to our institution, further examinations were completed. On 9 October 2024, ultrasound-guided core needle biopsy of the right breast lesion (8–10 cores, including both the mass and diffuse microcalcifications within it) was performed. However, targeted biopsy of calcifications under mammographic or CT guidance was not specifically performed. Despite multiple sampling, sampling error could not be completely excluded[3]. Pathology revealed high-grade DCIS with focal suspicious microinvasion. Immunohistochemistry showed: ER (0%), PR (0%), HER2 (3 +), Ki-67 (40%), E-cadherin (+), p120 (−), CK5/6 (−), SMA (myoepithelial+), p53 (+, mutant pattern), and AR (3+, 60%) (Fig. 1A,B). This result was repeatedly reviewed and confirmed by 2–3 senior pathologists. Due to the inability to definitively diagnose microinvasion, the lesion was ultimately classified as “suspicious for microinvasion.” Core needle biopsy of the right axillary lymph node showed metastatic carcinoma consistent with breast origin. Immunohistochemistry revealed ER (0%), PR (0%), HER2 (3+), Ki-67 (50%), CK5/6 (−), p63 (−), AR (90%, 2 +), E-cadherin (+), p120 (−), p53 (+, mutant pattern), GATA-3 (+), and PCK (+) (Fig. 2A,B). Cervical ultrasound and magnetic resonance imaging (MRI) also revealed an abnormal right subclavian lymph node. However, biopsy was not performed because the node was located deep and adjacent to major vessels. After multidisciplinary discussion, considering the technical difficulty and potential risks, we chose close clinical and imaging follow-up rather than invasive biopsy. Positron emission tomography/computed tomography (PET/CT) was considered but not performed due to cost. Therefore, the abnormal nature of the subclavian lymph node lacked histopathological confirmation.

The patient was clinically diagnosed with right breast cancer with right axillary lymph node metastasis and suspected right subclavian lymph node metastasis, and NAT was indicated. Considering both disease status and insurance coverage, the treatment regimen was determined as EC × 4 cycles followed by T × 4 cycles combined with trastuzumab and pertuzumab. The patient’s height was 162 cm, weight 46 kg, and body surface area 1.44 m^2^. The chemotherapy doses were as follows: Epirubicin 90–100 mg/m^2^ × 1.44 = 129.6–144 mg (administered as 140 mg); Cyclophosphamide 600 mg/m^2^ × 1.44 = 864 mg (administered as 860 mg). Both were given every 3 weeks. Trastuzumab was administered at a loading dose of 8 mg/kg, followed by 6 mg/kg every 3 weeks. Pertuzumab was administered at a loading dose of 840 mg, followed by 420 mg every 3 weeks. Dual anti-HER2 therapy was given concurrently with all 4 cycles of docetaxel (T), followed by completion of 1 year of therapy postoperatively. Skin tattooing at the primary tumor site was routinely performed before NAT for localization. The patient completed all 8 cycles of treatment between October 2024 and March 2025. The interval between the last cycle and surgery was 2 weeks. No dose delays or significant toxicities (such as radiation recall dermatitis or toxic epidermal necrolysis) were observed^[^4–6^]^.

**Figure 3.**
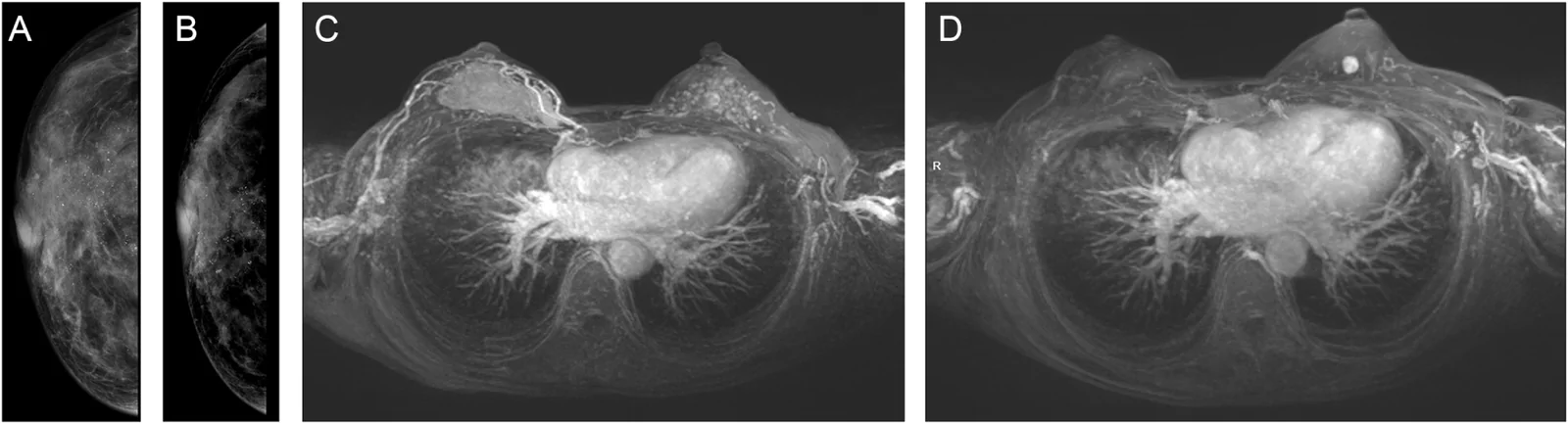
Pre-NAT and post-NAT imaging comparison. (A) Pre-NAT mammogram showing a mass with clustered microcalcifications. (B) Post-NAT mammogram showing a marked reduction in the mass, while calcifications persisted. (C) Pre-NAT breast MRI showing a large, irregular, enhancing lesion in the right breast and enlarged axillary lymph nodes. (D) Post-NAT breast MRI showing near-complete disappearance of the original enhancing lesion, with only a few punctate residual enhancements; the enlarged lymph nodes are no longer visible.

Response evaluation during and after NAT showed marked regression. Breast and axillary ultrasonography demonstrated a reduction of the right breast lesion from 8.90 × 1.74 cm to 1.99 × 1.62 × 1.12 cm, and previously enlarged axillary nodes were no longer visible after NAT. Cervical ultrasonography showed that the abnormal right subclavian lymph node, which had measured approximately 1.54 × 0.62 cm before NAT, also became undetectable. Although histological confirmation was lacking, its favorable response to NAT strongly suggested that it was indeed pathological. Pre-NAT mammography showed a right breast mass measuring approximately 2.36 × 2.00 cm with clustered punctate calcifications (BI-RADS 4C) (Fig. 3A). After NAT, the mass decreased to approximately 1.95 × 1.86 cm, but the calcifications persisted (BI-RADS 4C) (Fig. 3B). Stereotactic biopsy of the calcifications had been considered before NAT but was not performed due to patient preference and the presence of a clear mass. After NAT, biopsy was not performed because mastectomy was planned. Breast MRI demonstrated an enhancing lesion measuring approximately 28 × 73 × 80 mm before NAT, which almost completely disappeared after treatment, leaving only a few punctate foci of enhancement. Enlarged axillary lymph nodes were no longer visualized (Fig. 3C,D). No other abnormalities were identified on systemic evaluation.

After a comprehensive preoperative assessment excluded surgical contraindications, the patient underwent a modified radical mastectomy of the right breast with right axillary, right subclavian, and right interpectoral lymph node dissection under general anesthesia on 31 March 2025. Mastectomy was selected based on the following considerations: (1) Clinical experience suggests that extensive residual microcalcifications on mammography are often associated with residual tumor, posing an oncological risk for breast-conserving surgery. (2) The patient had a small and flat breast, with the tumor located centrally, making cosmetic outcomes unfavorable. (3) The patient preferred mastectomy to avoid reoperation and residual disease. (4) The decision was made after a thorough discussion of risks and benefits. Postoperative pathology showed complete disappearance of the tumor component in the right breast, including the DCIS component (Fig. 1C). Among the dissected lymph nodes, 0/2 right subclavian nodes and 0/19 right axillary nodes contained metastasis, and the right interpectoral specimen consisted of adipose tissue only (Fig. 2C). Overall, Miller–Payne grading indicated pCR. Extensive pathological sampling of the tumor bed was performed, including the tattoo-marked region, upper quadrant, and all surgical margins (nipple, skin, deep, superior, inferior, medial, and lateral), with a total of 50 paraffin blocks obtained. Serial sectioning was carried out to minimize the risk of missing residual DCIS or invasive carcinoma.

The patient subsequently completed 1 year of trastuzumab/pertuzumab therapy and received postoperative radiotherapy. At the latest follow-up, she remained in good general condition, with no evidence of recurrence or metastasis.

## Discussion

### Reappraising the phenomenon: from “DCIS sensitivity” to the bystander effect

Because of its protective basement membrane, relatively sparse microvasculature, and lower proliferative state, DCIS has historically been regarded as insensitive to NAT^[^7,8^]^. More recently, however, several studies have reported regression or even complete disappearance of DCIS after NAT^[^9–11^]^. In the present case, the DCIS component completely disappeared after treatment. Does this mean that DCIS itself is chemosensitive? Probably not. A key feature of this case is the presence of focal suspicious microinvasion on biopsy. Although the pathological results were repeatedly reviewed and confirmed by two to three senior pathologists, the presence of multiple axillary and suspected supraclavicular lymph node metastases cannot be ignored. It is undeniable that core needle biopsy may have underestimated the extent of disease involvement[12]. However, the possibility of pure DCIS with no invasion cannot be completely ruled out, as studies have shown that DCIS may also have a certain rate of lymph node metastasis[13]. Therefore, the lesion was ultimately classified as “suspicious for microinvasion.” While microinvasion was not definitively diagnosed, even subtle structural disruption may have pharmacologic significance, further strengthening the likelihood of a bystander effect. Additionally, studies indicate that at least 20% of patients initially diagnosed with DCIS by core needle biopsy eventually show invasive carcinoma on final pathology[3]. However, the natural progression of carcinoma cells from DCIS to DCISM and ultimately to invasive ductal carcinoma remains unclear[14].

DCISM is a distinct entity accounting for approximately 0.6–3.4% of all breast cancers^[^15,16^]^. It is defined as DCIS with extension beyond the basement membrane, with the largest invasive focus measuring ≤1 mm. This minute structural breach may have major pharmacologic implications: it is analogous to opening a small gap in an otherwise intact dam. Through this gap, high concentrations of chemotherapeutic agents may diffuse from the surrounding vascular bed into the ductal lumen, thereby exerting a bystander killing effect on adjacent DCIS cells[17]. In other words, the disappearance of DCIS in this setting is more plausibly explained by passive drug diffusion after disruption of the physical barrier than by active uptake and intrinsic sensitivity of DCIS cells to cytotoxic therapy. Additionally, the tumor microenvironment plays a crucial role^[^2,18^]^. The myoepithelial layer and basement membrane form a drug-exclusion barrier, while microinvasion disrupts this barrier, allowing drug penetration and potentially inducing immune cell infiltration and stromal signaling changes, thereby promoting tumor regression. Furthermore, biomarkers such as STAT3[19] and ENPP1[20] have been suggested as potential early detection markers for breast cancer; however, these markers are not routinely assessed in our institution. At present, there is still no consensus on whether DCISM should be managed more like DCIS or invasive carcinoma^[^21,22^]^. If microinvasion truly existed in this case, it could have provided a direct pathway for drug diffusion. If not, the complete response suggests either the presence of unsampled invasive carcinoma or, less likely, intrinsic chemosensitivity of HER2-positive DCIS.

### Diagnostic recalibration: the limitations of core needle biopsy

DCIS with lymph node metastasis is uncommon, with reported rates ranging from 1.8% to 2.9%^[^23,24^]^. In contrast, lymph node metastasis occurs in approximately 8–9% of DCISM cases[25]. Previous studies have shown that, compared with pure DCIS, DCISM is more often associated with high nuclear grade, HR negativity, HER2 positivity, and high Ki-67 expression, all of which are linked to a greater risk of nodal metastasis^[^26,27^]^. In the present case, core needle biopsy suggested DCISM, and axillary nodal metastasis was confirmed. The suspicious right subclavian node was not biopsied because of technical limitations. Ultrasound- or CT-guided core needle biopsy could have been considered, and PET/CT might have provided supportive evidence of metastatic disease involvement to strengthen staging[28]. The absence of histopathological confirmation represents a limitation, precluding definitive classification of the subclavian lymph node as metastatic. Nevertheless, its complete disappearance after NAT strongly suggests underlying pathological involvement.

Image-guided core biopsy (CT-guided or ultrasound with experienced interventional radiology) or PET/CT could have been pursued preoperatively to strengthen staging.

Even with adequate sampling, an invasive component may still have been missed by biopsy. Therefore, disappearance of the DCIS component after NAT may represent a form of reverse pathological calibration: the effectiveness of NAT implies that an unsampled invasive component was likely present, and once the invasive carcinoma was eradicated, the associated DCIS may also have disappeared through a bystander effect. Put differently, loss of the DCIS component may indirectly support the prior existence of invasive disease. Although pure DCIS may very rarely be associated with lymph node metastasis, the likelihood of an occult invasive component rises substantially when nodal metastasis is present.

### Imaging dilemmas: how should residual microcalcifications be interpreted?

Mammography is the main imaging modality for detecting DCIS and DCISM. However, its value in assessing residual disease after NAT is limited, largely because the nature of post-treatment microcalcifications is difficult to determine[29]. In the present case, residual microcalcifications remained visible on mammography after NAT (Fig. 2B), whereas postoperative pathology confirmed pCR with the disappearance of the DCIS component (Fig. 1B). This finding further illustrates the limitations of inferring residual DCIS solely from persistent calcifications.

Available evidence suggests that residual microcalcifications after NAT may represent benign changes, necrotic tissue, or stromal reaction within the tumor bed rather than viable tumor cells[30]. Current routine imaging techniques cannot reliably distinguish DCIS-related calcifications from benign or treatment-related calcifications. Residual microcalcifications, therefore, do not necessarily indicate residual DCIS, and they do not always show obvious enhancement on MRI[31]. In this patient, stereotactic biopsy of the calcified area was not performed before NAT, so the histologic nature of the calcifications could not be confirmed. They may have represented DCIS, calcifications within invasive ductal carcinoma, or preexisting benign calcifications. After learning the postoperative pathology results, the patient remarked that breast-conserving surgery might have been possible. This experience underscores how reliance on the extent of residual microcalcifications alone may lead to imaging-driven overtreatment[32], such as mastectomy or unnecessarily wide excision, even when no viable tumor remains.

For patients who show a marked systemic response to NAT – such as nodal conversion to negative status and disappearance of MRI enhancement – residual microcalcifications should not be used as the sole determinant of surgical extent. A more cautious strategy may include (1) multimodal imaging assessment, combining MRI functional sequences such as diffusion-weighted imaging and dynamic contrast-enhanced patterns with ultrasonographic elastography^[^33–35^]^; (2) targeted stereotactic biopsy of calcified areas, with consideration of breast-conserving surgery if no residual tumor is identified pathologically^[^36,37^]^; and (3) image-guided localization of calcifications when breast conservation is pursued, for example, with preoperative mammographic localization or radioactive seed placement to ensure precise excision. Until stronger guideline-based evidence becomes available, management of these calcified areas will remain a clinical challenge.

### Limitations

As a single case report, this study has several limitations. First, conclusions from an individual case cannot be generalized without caution. Second, the calcified area was not biopsied after NAT, so its exact nature could not be confirmed. Third, the suspicious subclavian lymph node lacked pathological confirmation. Finally, the follow-up period is still limited, and long-term outcomes require further observation.

## Conclusion

The value of this case does not lie in proving that DCIS is sensitive to NAT. Rather, it highlights a clinically important diagnostic pitfall and may help refine individualized treatment strategies. When pre-NAT biopsy shows DCIS/DCISM with nodal disease, perform aggressive efforts to detect occult invasion (repeat/core rebiopsy, image-guided nodal sampling, PET/CT) and plan for targeted biopsy of residual calcifications before committing to mastectomy when clinical response is robust. Future prospective studies are needed to establish more precise diagnostic approaches for such phenotypically discordant cases, potentially integrating radiomics, liquid biopsy, or repeat biopsy techniques.

## Data Availability

Available.

## Reporting guideline

This case report was prepared in accordance with the SCARE 2025 guideline[38].
